# Editorial: AI-driven smart sensing and processing for personalized healthcare

**DOI:** 10.3389/fmed.2026.1820423

**Published:** 2026-07-07

**Authors:** Wei Wang, Lewei Zhao, Junxin Chen

**Affiliations:** 1 Sun Yat-sen University, Guangzhou, China; 2 MedStar Georgetown University Hospital, Washington, DC, United States; 3 Dalian University of Technology, Dalian, China

**Keywords:** AI for healthcare, artificial intelligent of thing (AIoT), medical big data, smart healthcare, smart sensing

The convergence of artificial intelligence (AI), smart sensing technologies, and advanced data processing is reshaping the landscape of personalized healthcare (1). Continuous physiological monitoring (2), intelligent imaging systems (3), wearable devices (4), and scalable computational models now enable individualized assessment and intervention at unprecedented resolution (5, 6). This Research Topic, *AI-driven smart sensing and processing for personalized healthcare*, brings together interdisciplinary contributions that advance both the methodological foundations and translational applications of AI-enabled health technologies.

A prominent theme across this Research Topic is the movement of intelligence closer to the point of care. Rather than relying solely on centralized computing infrastructure, several studies demonstrate how AI models can operate efficiently on edge devices. Berki et al. explore the feasibility of real-time, on-device ECG biometric classification using quantized neural networks. By reducing model complexity while preserving classification performance, their work highlights how computational efficiency, privacy preservation, and low-latency inference can coexist in wearable and mobile health systems. Such advances are critical for secure, continuous cardiac monitoring and identity authentication in real-world settings.

Remote and accessible rehabilitation assessment represents another major direction. Unger et al. demonstrate the feasibility of detecting upper limb compensation in individuals post-stroke using deep learning applied to consumer-grade webcams. This approach lowers barriers to movement analysis by replacing specialized motion-capture systems with widely available hardware. Complementing this work, Tran et al. apply deep learning techniques to accelerometry data to evaluate upper extremity movement in stroke survivors. Together, these contributions illustrate how multimodal sensing combined with AI-driven analysis can enable scalable, home-based neurorehabilitation and personalized recovery monitoring.

Advances in intelligent medical imaging are also well-represented. Norouziazad et al. propose a robust colonoscopy polyp segmentation framework incorporating dynamic-Nu T-Loss, multi-scale learning, and uncertainty-aware adaptation. Accurate polyp detection and segmentation are essential for early colorectal cancer prevention. By explicitly modeling uncertainty and adapting to variability in endoscopic imagery, this work addresses key challenges in real-world clinical deployment. It reflects a broader methodological shift toward reliability-aware AI systems that acknowledge and manage ambiguity in medical data.

In the behavioral and neurodevelopmental domain, Al-Adhaileh et al. present a deep learning approach for diagnosing autism spectrum disorder using eye-tracking technology. Eye movement patterns provide rich behavioral biomarkers, and AI-driven pattern recognition enhances the ability to detect subtle gaze dynamics. This study exemplifies how smart sensing, when paired with advanced computational modeling, can support earlier and more objective screening tools in complex conditions where traditional assessments may be subjective or resource-intensive.

The Topic also includes important review and perspective contributions that situate technical innovation within broader healthcare contexts. Gill et al. review the role of artificial intelligence in managing posterior capsule opacification, synthesizing current developments and outlining future directions in ophthalmologic care. Their work underscores the growing integration of AI-assisted analysis into clinical decision-making and treatment planning.

Sun et al. examine the potential of digital twin technology to transform public health emergency management within the PPRR framework. By envisioning dynamic, data-driven replicas of populations and systems, digital twins offer a bridge between personalized data streams and macro-level health governance. This perspective expands the concept of personalization beyond the individual, suggesting how AI-driven sensing can inform adaptive, system-wide responses to public health challenges.

Human factors are equally critical in realizing the promise of digital transformation. Andersson and Gonzalez highlight digital health literacy as a foundational enabler of effective AI adoption. Their perspective reminds us that technological sophistication must be matched by user understanding, equitable access, and trust. Without attention to these dimensions, even the most advanced AI systems risk underutilization or widening disparities.

Finally, Song and Liu provide an international visualization analysis of research hotspots and trends in clinical decision support systems using CiteSpace. By mapping the intellectual structure of this rapidly evolving field, their work contextualizes AI-driven smart sensing within the broader ecosystem of data-driven clinical support. Such meta-analyses help identify emerging directions, collaborative networks, and future opportunities for innovation.

Across these contributions, several cross-cutting insights emerge. First, edge computing and resource-aware AI are becoming central to scalable, privacy-conscious healthcare delivery. Second, multimodal sensing integration enhances the robustness and richness of personalized assessments. Third, uncertainty-aware modeling is increasingly recognized as essential for safe and reliable clinical deployment. Fourth, personalization spans multiple levels, from individual physiology and behavior to population-scale digital twins. Finally, technological progress must be accompanied by attention to literacy, ethics, and equitable implementation.

As AI-driven smart sensing systems continue to mature, interdisciplinary collaboration remains vital. Engineers, clinicians, data scientists, and public health experts must work together to ensure that innovations are not only technically sound but also clinically meaningful, ethically governed, and socially inclusive. The studies collected in this Research Topic demonstrate tangible progress toward intelligent, context-aware, and patient-centered healthcare systems.

We extend our sincere appreciation to all authors, reviewers, and editors who contributed to this Research Topic. Their collective efforts highlight the transformative potential of AI-driven smart sensing and processing in advancing personalized healthcare.
